# Vulnerability to Substance Abuse and the Risk of Suicide in Students of Region 12 of Islamic Azad University

**DOI:** 10.5812/ijhrba.11229

**Published:** 2014-06-15

**Authors:** Nader Monirpoor, Helen Khoosfi, Morteza Gholamy Zarch, Mohsen Tamaddonfard, Seyed Farzad Tabatabaei Mir, Maryam Mohammad Alipour, Yasamin Karimi

**Affiliations:** 1 Department of Clinical Psychology, Qom Branch, Islamic Azad University, Qom, IR Iran; 2 Counseling Center, University of Shahed, Tehran, IR Iran; 3 Young Researches Club, Rudehen Branch, Islamic Azad University, Rudehen, IR Iran; 4 Young Researches Club, Qom Branch, Islamic Azad University, Qom, IR Iran; 5 Department of Clinical Psychology, Arak Branch, Islamic Azad University, Arak, IR Iran; 6 Department of Clinical Psychology, Markazi Sciences and Research Branch, Islamic Azad University, Arak, IR Iran; 7 Department of Counseling, University of Shahid Beheshti, Tehran, IR Iran

**Keywords:** Vulnerability to Substance Abuse, Risk of Suicide, Students

## Abstract

**Background:**

Substance abuse prevalence and the number of suicides among university students is less than public population; however the sensitivity of society regarding the occurrence of such damages among students puts special emphasis on appraising these variables. More than 30% of Iranian students study in Islamic Azad University.

**Objectives:**

The current research aimed to appraise the vulnerability of substance abuse and the risk of suicide in students of region 12 of Islamic Azad University.

**Patients and Methods:**

In the current study, 1053 students (606 boys and 447 girls) with the average age of 22.55 years were selected through stratified sampling from Karaj, Takestan, Qazvin and Qom branches of Islamic Azad University. In order to assess the variables, Mental Health Worksheet of Central Counseling Office of the Ministry Science, Research and Technology was utilized.

**Results:**

Average, standard deviation, minimum and maximum scores in substance abuse vulnerability of the students in region 12 were measured as 36.28, 14.68, 11.22 and 92.87; and the same for risk of suicide were 31.29, 15.61, 7.93 and 96.30, respectively. Students in Qom branch were significantly less vulnerable to substance abuse and less exposed to the risk of suicide than their peers in Karaj, Qazvin and Takestan branches.

**Conclusions:**

Less significant possibility of substance abuse and risk of suicide in students of Qom branch in comparison with other branches could be due to numerous variables particularly their religious attitudes. Nevertheless the average of these variables among the students of region 12 were higher than the reported scores of their peers in the state universities which reflects the serious need for precise assessments and providing preventive services and mental health interventions.

## 1. Background

University Students hold a special position in each society and occurrence of any serious psychological problem among them can expose the society to considerable problems (1), Substance abuse and suicide are among psychological problems of the university students (2). Miscellaneous studies have reported the incidence of abuse and dependency on substances among university student population (3). For instance, a research on the students of Midston University indicated that 17% of the boys and 11% of the girls use unauthorized substances and stimulant drugs (4). Appraising the undergraduate students of Iran, the prevalence of substance abuse longevity among them was reported as follows: hookah 30%, cigarettes 20%, alcoholic drinks 13%, opium 2.8%, hashish 1.8%, ecstasy pills 0.8%, crystal 0.6%, crack 0.4%, heroin 0.3% (5). Besides, using the questionnaire of vulnerability to substance use in another study, average, standard deviation, minimum and maximum vulnerability of newly admitted students in the state universities were measured as 26.18, 3.92, 17 and 55, respectively (2).

In addition to substance abuse, risk of suicide also exists in students’ society and it is the second reason of death among them (6). The rate of students suicide is 7.5 in 100,000 individuals, which is almost half the rate of suicide among their non-students peers (7, 8). A study in Iran (9) indicated that the number of suicides including suicide attempts and completed suicides 1998 to 2004 was 292 cases, among which 25 instances (8.6%) were completed suicides and 265 cases (90.8%) were suicide attempts, and 48.9% of the cases were boys and the rest 51.1% were girls. In another study, frequency of students’ suicide from 2004 to 2006 was reported 268 cases, including 94 (35.1%) women and 174 (64.9%) men. Tehran with 34 cases (12.6%) was followed in the first city with suicide instances and Kermanshah, Azarbaijan, Mazandaran, Gilan, Fars, Isfahan and Ilam followedIn a countrywide study, using the vulnerability to suicide questionnaire, on Iranian students, average, standard deviation, minimum and maximum vulnerability to suicide were reported as 20.45, 4.7, 11 and 48 respectively (2).

Islamic Azad University, possessing 18 regions and around 400 university branches, is considered as one of the biggest universities of the world which covers approximately 30% of Iran’s university students in various university levels and different fields of study. However, researches concerning the assessment of vulnerability to substance abuse and risk of suicide among student sample of Islamic Azad University have been insufficient and there is an evident lack of a screening and comprehensive research in such fields, at least in one of the 18 regions of Islamic Azad University.

## 2. Objectives

The current research aimed to appraise the rate of vulnerability to substance abuse and the risk of suicide in the students of region 12 of Islamic Azad University.

## 3. Patients and Methods

### 3.1. Participants and Plan

The current study was a survey and descriptive research. Research samples included 1053 university students (606 boys and 447 girls), with the average age of 22.55 years selected through stratified sampling from Karaj, Takestan, Qazvin and Qom branches of Islamic Azad University.

### 3.2. Instruments 

#### 3.2.1. Scale of Vulnerability to Substance Abuse

In order to assess the rate of vulnerability to substance abuse among the students, the Students’ Mental Health Worksheet scale of the Ministry of Science was applied (10). This questionnaire consists of 87 items and questions 41 to 59 measures the vulnerability to substance abuse and dependency. These questions were prepared by reviewing reliable scientific resources regarding the most significant factors of individual’s vulnerability to substance abuse and also two questionnaires of American Drug and Alcohol Survey and Prevention Planning Survey. The scale of substance abuse vulnerability has acceptable construct validity and each of its questions indicates one risk factor which has widespread scientific support (2, 10, 11). Alpha coefficient of this scale was calculated as 0.52 in the 1053 students’ sample of Islamic Azad University.

#### 3.2.2. Risk of Suicide Scale

In order to assess this variable, Students’ Mental Health Worksheet of the Ministry of Science was utilized (10). The questions 21 to 40 are used as a scale for assessing the risk of suicide. To prepare this scale, in addition to posing questions regarding the most important factors in suicide risk, questions from the two questionnaires of treatment opportunities and young people screening plan and also young people screening questionnaire were selected and the construct validity of this scale was reported acceptable (2, 10, 11). Alpha coefficient of this scale was calculated as 0.66 in the 1053 student sample of Islamic Azad University.

## 4. Results

Analyzing the risk factors of substance abuse (questionnaires queries) indicated that 20.4% of students had experienced smoking cigarettes throughout their lives, and 15.5% of them smoked cigarettes at the moment. Besides, 41.3% of them declared using hookah in their lifetime, among which 32.9% were users at the moment, 6% of the students reported no control from the side of the family, 49.3% restricted control and 44.7% constant control of the family on their behaviors; 43.1% expressed that they sometimes had to be in contact with someone who uses alcoholic drinks and 13.9% of them reported having constant relationship with such people; 33.5% stated that they sometimes had to be in touch with someone who uses drugs and 4.2% declared having constant contact with drug users; 33.8% reported that they had sometimes observed sales and purchase of drugs in their living place and 7.1% had regularly observed it; 12.4% sometimes and 1.7% regularly used sedatives; 56.1% believed that everything is worth trying once. Concerning impulses and desires control, 6.4% declared having no control, 42.1% limited control, and 23.6% reported on and off control. Regarding the capability to object and to say no, 4.6% declared being incapable, 35.9% restricted capability and 24.4% stated on and off capability; 15.8% of the students introduced themselves as being most of the times risk-taking, 35% sometimes risk taking and 38.9% to some extent risk taking. Regarding self-confidence, 10.9% of the students declared lack of self-confidence, 36.5% limited self-confidence and 27.6% stated being sometimes self-confident; 44% of the students expressed having close friends who smoke; 28.1% of students’ fathers, 2.7% of their mothers, 1.5% of sisters and 10.9% of brothers smoked cigarettes; 16.2% of students reported that their parents’ relationship is argumentative, 1.3% of their parents lived separately and 1.3% of their parents were divorced; 8.2% of the fathers and 2.8% of mothers had passed away.

Average, standard deviation, minimum and maximum scores of vulnerability to substance abuse among the students of region 12 of Islamic Azad University were calculated as 40, 14.70, 13.22 and 96.21, respectively. In addition, the results of the one-way variance analysis (P < 0.001, df = 3, F = 12.25) indicated significant difference between the students from different branches of the region 12 of Islamic Azad University regarding vulnerability to substance abuse (Table 1). The results of Scheffe test demonstrated that this difference was between the students of Qom and Karaj branches. As a matter of fact the students of Qom Branch were significantly less vulnerable than their peers in Karaj Branch (Table 2).

Besides, results of the one-way variance analysis (P < 0.05, df = 3, F = 2.62) indicated a significant difference between the students of region 12 branches regarding the risk of suicide (Table 3). The results of Scheffe test indicated that this difference was between Qom and Karaj branches. In fact students of Qom Branch were significantly less vulnerable to suicide than the students of Karaj Branch.

**Table 1.** Number, Average and Standard Deviation of Vulnerability to Substance Abuse in Students by University Branch ^a^

University Name	Number	Results
**Takestan**	196	41.10 ± 13.77
**Qazvin**	334	40.76 ± 14.68
**Karaj**	398	41.14 ± 15.15
**Qom**	125	32.65 ± 12.60

^a^ Data are presented as Mean ± SD.

**Table 2.** Number, Average and Standard Deviation of Suicide Risk in Students by University Branch ^a^

University Name	Number	Results
**Takestan**	196	30.71 ± 15.82
**Qazvin**	334	31.62 ± 15.12
**Karaj**	398	32.33 ± 16.48
**Qom**	125	27.97 ± 13.53

^a^ Data are presented as Mean ± SD.

In addition, results of independent groups t-test showed no significant difference between boys and girls concerning the risk of suicide (P < 0.761, t = 0.30), nevertheless boys were more vulnerable to substance abuse (P < 0.001, t = 8.31). There was no significant difference between single and married students regarding suicide risk (P < 0.50, t = 0.67), but single students were more vulnerable to substance abuse (P < 0.01, t = 2.59). No significant difference was found between part-time and full-time students concerning the risk of suicide (P < 0.49, t = 0.68), however part-time students were more vulnerable to substance abuse (P < 0.039, t = 2.06). No significant difference in vulnerability to substance abuse (P < 0.71, t = 1.17) and risk of suicide (P < 0.21, t = 1.24) was observed among the self-administered students and the rest with families of martyrs and war disabled persons. Additionally, comparison of students from various regions regarding the risk of suicide indicated no significant difference between the ones living with families, those in dormitories, students living with friends in rented houses and students living alone in rented houses (P < 0.42, df = 3, F = 0.93). However students living with friends in rented houses were more vulnerable to substance abuse in comparison to those living with families and the ones in dormitories (P < 0.001, df = 3, F = 5.80) (Table 3).

**Table 3.** Number, Mean and Standard Deviation of Suicide Risk in Students by Demographic Variables ^a^

	Number	Vulnerability to Substance Abuse	Risk of Suicide
**Boys**	606	43.14 ± 15.59	31.16 ± 16.15
**Girls**	447	35.75 ± 12.18	31.46 ± 14.95
**Single**	859	40.22 ± 14.76	31.25 ± 15.52
**Married**	116	36.47 ± 13.53	30.21 ± 15.79
**Full Time**	937	39.71 ± 14.57	31.17 ± 15.68
**Part Time**	56	43.92 ± 16.33	32.64 ± 16.24
**Self-administered students**	758	40.00 ± 14.75	30.99 ± 15.10
**Students with families of martyrs and disabled war scarifies**	51	40.79 ± 13.23	33.72 ± 16.22
**Living with family**	889	39.48 ± 14.05	31.25 ± 15.66
**Living in dormitory**	64	39.85 ± 18.31	29.49 ± 14.56
**Living with friends in rented houses**	56	47.37 ± 15.72	33.00 ± 15.71
**Living alone in rented houses**	18	44.81 ± 20.27	35.49 ± 21.24

^a^ Data are presented as Mean ± SD.

## 5. Discussion

The present research aimed to appraise the level of vulnerability to substance abuse and the risk of suicide of the students in region 12 of Islamic Azad University. The results of this study regarding smoking cigarettes (15.5%) and hookah (32.9%), almost corresponded with the outcomes of the study implemented by Sohrabi et al. (5) which reported smoking cigarettes and hookah to be respectively 20% and 30% in bachelors’ students of the state universities.

Students of region 12 of Islamic Azad University shad higher average, standard deviation, minimum and maximum scores in vulnerability to substance abuse (40, 14.70, 13.22 and 96.21, respectively) than the students of the state universities (2) (26.18, 3.92, 17 and 55, respectively). Students of region 12 gained higher scores in average, standard deviation, minimum and maximum of suicide risk (31.29, 15.64, 7.93 and 96.30, respectively) in comparison to students of the state universities (2) (20.45, 4.76, 11 and 48, respectively). Such differences between the students of region12 and the state universities could be driven from their distinct dominating circumstances like paying the tuition, less psychological support for the students of Islamic Azad University and other influential factors. Students of the state Universities not only pay lower tuitions and benefit from inexpensive dormitories, but they also receive free psychological support from the universities’ counseling centers. Whereas students of the Islamic Azad University, in addition to paying high tuitions, do not receive the least psychological support and experience more psychological, social, educational, familial and economical stressors in comparison to state university students (12).

The comparison of the students in region 12 indicated that students of Qom branch scored lower average regarding vulnerability to substance abuse and risk of suicide than their peers in Takestan, Qazvin and Karaj branches, and this difference was significant compared to the students of Karaj branch. Although this difference could result from various grounds, it appears that cultural context together with religious learnings of the students in Qom branch play significant roles for their being less vulnerable to substance abuse and risk of suicide, since most of the studies (13-16) have confirmed the preventive influence of religion in psychological disorders and problems such as suicide and substance abuse. Besides, religion has presented numerous inhibitive orders regarding self-preservation.

Alike the study of the Ministry of Science (2), in the present study there was no significant difference regarding the risk of suicide between boys and girls and between married and single students; however boys and singles were respectively more vulnerable to substance abuse in comparison to girls and married ones. Additionally these findings are consonant with the outcomes of some studies (5, 10, 17, 18). Besides, the 4th revised edition of diagnostic and statistical manual of mental disorders (19) reports that substance abuse in men and singles are higher than women and married people. This could be due to gender variables, inhibiting cultural beliefs for women, easier access to substances for men and more social support for married individuals in comparison to singles. In line with the outcomes of the implemented studies on the students of the state universities (2, 5, 10) , the present research also demonstrated that residing with friends in a rented house would increase students’ vulnerability to substance abuse because of less control of the family, more pressure of the peer group, availability of inappropriate behavioral models and other risk factors. While living with family and in dormitories would decrease vulnerability to substance abuse due to the existence of various controls and receiving psychological and emotional support from family, friends and university authorities.

The present study confronted some restrictions, such as distribution of statistical society of the students in region 12 of Islamic Azad University and heterogeneous classification of students in universities with comprehensive ratings of independent, grand, small and college and this prevented the inclusion of the students from all branches and colleges in the sampling. Besides, in the current study the questionnaires of vulnerability to substance abuse and the risk of suicide were utilized and although they consisted of risk factors, they did not present multidimensional assessment of the related variables and merely confined to the total score of vulnerability. Therefore the authors suggest including all the students of the branches and colleges of region 12 in sampling for further researches on this issue and also using assessment scales for substance abuse, vulnerability, and suicide risk which consist of miscellaneous subscales and present their multidimensional portrait. Additionally, the present study suggests the authorities of Islamic Azad University to help increase the psycho-social control and support of the families through expanding native selection policies and consequently decrease substance abuse, vulnerability, and suicide. The authorities can also promote provision of psychological services to students through benefitting from high capacity of recruited psychological and counseling experts of the universities. Finally, allocating research budgets to the issues concerning students’ mental health provides the grounds for precise determination and screening of high-risk students.

## References

[1] Monirpoor N, Khoosfi H, Yaghoubi H (2009). Students’ mental health; community-based attitude and peer counselors..

[2] Yaghoubi H, Akbari Zardkhaneh S, Vaghar Anzabi M (2008-09). Report of students’ mental health status in the universities of science.

[3] Poursharifi H, Bolhari J, Peyravi H, Yaghubi H, Zarani F, Hafezi M (2009). Report of the first phase of the pilot study of comprehensive plan of suicide prevention in the University of Tehran.. J Res Behave Sci..

[4] Hall KM, Irwin MM, Bowman KA, Frankenberger W, Jewett DC (2005). Illicit use of prescribed stimulant medication among college students.. J Am Coll Health..

[5] Sohrabi F, Akbari Zardkhaneh S, Taraghijah S, Falsafinezhad MR, Yaghoubi H, Ramezani VA (2009). Substance Abuse Among State University Students, Iran, 1385-1386.. Soc Welf..

[6] Haas AP, Hendin H, Mann JJ (2003). Suicide in college students.. Am Behav Sci..

[7] Barrios LC, Everett SA, Simon TR, Brener ND (2000). Suicide ideation among US college students. Associations with other injury risk behaviors.. J Am Coll Health..

[8] Joffe P (2008). An empirically supported program to prevent suicide in a college student population.. Suicide Life Threat Behav..

[9] Zarani F, VagharAnzabi M (2006). Appraising epidemiology of students’ suicide..

[10] Poursharifi H, Taremian F, Zarani F, VagharAnzabi M, Jafari A (2005). The plan of investigating new entrants’ mental health in the universities of science, research and technology universities during educational year of 2004-05..

[11] Taraghijah S, Sohrabi F, Najafi M, Falsafinejad MR (2010). Assessment of Iranian Students'mental Health and It's Correlation with Some Psychological Variables in Year 85-86.. Couns Res Develop..

[12] Rabiei F, Kafi SM, Moosavi SS, Baradaran M (2012). Stressors of the students in Gilan’s Islamic azad, state and Payam-Nour universities..

[13] Bergin AE (1983). Religiosity and mental health: A critical reevaluation and meta-analysis.. Prof Psychol Res Pr..

[14] Bahrami Ehsan H, TahbazHoseinzadeh S (2007). The dimensions of relation between religious orientation and mental health and assessing scale of religious orientation.. J Psychol Educ Sci..

[15] HasaniVajari K, BahramiEhsan H (2005). The role of religious confronting and spiritual fontune in determining mental health.. J Psychol Educ Sci..

[16] Sharifi T, MehrabizadehHonarmand M, Shekarkan H (2005). Religious attitude and general health and patience in students of Islamic Azad university of Ahwaz.. Thought behav..

[17] Taremian F, Bolhari J, Pairavi H, Ghazi Tabatabaeii M (2008). The prevalence of drug abuse among university students in Tehran.. Iran J Psychiatry Clin Psychol ..

[18] Kordmirza E, Azad H, Eskandari H (2003). Normalizationof addiction potentialscale for spotting individuals exposed to drug abuse among the students of Tehran universities.. Rese Addict..

[19] Sadock BJ, Sadock VA (2007). Kaplan and Sadocks’ Synopsis of psychiatry behavioral..

